# Computer vision applied to herbarium specimens of German trees: testing the future utility of the millions of herbarium specimen images for automated identification

**DOI:** 10.1186/s12862-016-0827-5

**Published:** 2016-11-16

**Authors:** Jakob Unger, Dorit Merhof, Susanne Renner

**Affiliations:** 1 Institute of Imaging and Computer Vision, RWTH Aachen University, Kopernikusstr. 16, 52074 Aachen, Germany; 2 Systematic Botany and Mycology, University of Munich (LMU), Menzinger-Str. 67, 80638 Munich, Germany

**Keywords:** Automated identification, Computer vision, Herbarium specimens, JSTOR, Leaf shape, Leaf venation

## Abstract

**Background:**

Global Plants, a collaborative between JSTOR and some 300 herbaria, now contains about 2.48 million high-resolution images of plant specimens, a number that continues to grow, and collections that are digitizing their specimens at high resolution are allocating considerable recourses to the maintenance of computer hardware (e.g., servers) and to acquiring digital storage space. We here apply machine learning, specifically the training of a Support-Vector-Machine, to classify specimen images into categories, ideally at the species level, using the 26 most common tree species in Germany as a test case.

**Results:**

We designed an analysis pipeline and classification system consisting of segmentation, normalization, feature extraction, and classification steps and evaluated the system in two test sets, one with 26 species, the other with 17, in each case using 10 images per species of plants collected between 1820 and 1995, which simulates the empirical situation that most named species are represented in herbaria and databases, such as JSTOR, by few specimens. We achieved 73.21% accuracy of species assignments in the larger test set, and 84.88% in the smaller test set.

**Conclusions:**

The results of this first application of a computer vision algorithm trained on images of herbarium specimens shows that despite the problem of overlapping leaves, leaf-architectural features can be used to categorize specimens to species with good accuracy. Computer vision is poised to play a significant role in future rapid identification at least for frequently collected genera or species in the European flora.

**Electronic supplementary material:**

The online version of this article (doi:10.1186/s12862-016-0827-5) contains supplementary material, which is available to authorized users.

## Background

Global Plants, a collaborative between JSTOR and some 300 herbaria, is now the world’s largest database of digitized plant specimens (http://about.jstor.org/content/global-plants, accessed 20 April 2016). It currently contains images of 2,482,901 million herbarium sheets (Hannah Begley, Digital Librarian for Primary Sources, JSTOR, 4 May 2016). Each of the images is accompanied by metadata including the Latin (formal) species name and data transcribed from the label, such as name of collector (where known), collection date and location (where known), and the acronym of the herbarium that owns the specimen. Many of the images in JSTOR plants are of so-called type specimens, which have received priority in digitization projects because they are essential to biological nomenclature. As set out in the *Code of Nomenclature for algae, fungi, and plants* [1], type specimens fix the application of names, and by definition, a type specimen is, and always remains, correctly identified, no matter the changing taxonomic views and insights from new data. The plants shown in these images are not always perfectly preserved; for example, their leaves often overlap each other or are damaged.

Herbaria that are digitizing their type specimens are allocating considerable recourses to the maintenance of computer hardware and rental of storage space for growing numbers of images. It is therefore important to consider, and test, the future utility of the millions of high-resolution type specimens. An obvious desideratum in this context is the application of machine learning to quickly classify images into thousands of categories of interest to different user, such as “Rosaceae”, “*Crataegus*”, or “*Fagus sylvatica.*” So far, computer vision has not been applied to JSTOR plant specimen images. Instead, use of digitized plant images still relies exclusively on human pattern recognition and on the (excellent) memories of taxonomists who know under which Latin name to search for an image. Between June 2015 and April 2016, 282,403 unique visitors viewed 427,636 (17%) of the 2.48 million JSTOR plant images. During a slightly larger period of 1.5 years (Dec. 2014-April 2016), there were 419,822 unique visitors (Hannah Begley, Digital Librarian for Primary Sources, JSTOR, 4 May 2016). At this time, of course, only people who know the Latin name of a plant (or its synonymous names) can find images of specific species in JSTOR.

Machine learning applied to images of museum specimens offers the opportunity to identify (i.e., provide with a Latin name) specimens speedily, which would facilitate subsequent fine-scale analysis by taxonomic experts, for example, whether a specimens is an outlier in its traits or geographic range and may represent a new species. (Machines can never decide whether a specimen represents a new species because under the current *Codes of Nomenclature* species ranking is a matter of opinion). The application of machine learning and computer vision to museum specimens differs from applications of computer vision to living specimens where the primary goal is not find a Latin name for an unnamed specimen, but instead to cluster specimens for other purposes. So far, computer vision approaches in biology have been applied to cluster (images of) wings of *Drosophila* species [2], wings of common British moths [3], bee wings [4], and color spots of cichlid fish [5]. In plants, computer vision has been applied to images of 1907 fresh leaves belonging to 32 different plant species [6], images of fresh leaves of a few tree species [7], and images of fresh leaves of three legume species [8]. One study has applied machine learning to images of dead leaves, using (digitized images of) 7597 leaf clearings from 2001 genera of flowering plants to categorize leaf vein patterns [9]. Leaf clearings are leaves that have been chemically treated and preserved to show the veins. The images used in all these studies show non-overlapping leaves. Specimens in herbarium image databases, by contrast, have many overlapping leaves, presenting a challenge for the application of computer vision.

Here, we test whether a standard computer vision algorithm can be trained on images of typical herbarium specimens to learn to identify the 26 tree species (Additional file 1: Table S1) most commonly encountered in Germany (http://www.baumkunde.de/haeufigste-baeume-deutschland.php) and surrounding countries of Central Europe. Algorithms that examine morphometric characters usually require large training databases of images per category (here species). For example, Wilf et al. [9] used at least 100 images per category (fossil leaf morphotype = fossil leaf species). To generate a training database, we used only 10 images per species to simulate the empirical fact that most species are represented in image databases by just a few images. The photographed specimens were collected between 1820 and 1995 and were typical of herbarium material in often having overlapping leaves. Our approach to deal with the problem of overlapping leaves consisted of first segmenting single, non-overlapping leaves for each species in a preprocessing step. A normalization routine was then used to counteract distortions and ensure comparability of the images. Next, features of three categories were extracted from the normalized images and fed into a Support Vector Machine to achieve the final classification. No prior study has adopted this combination of tools for leaf identification, using herbarium material.

## Methods

### Imaging setup for herbarium specimens

To obtain the training images, we used the Munich herbarium’s HerbScan unit, which consist of a flatbed scanner (Epson Expression model 10000XL), modified for inverted use. Specimens were photographed at a resolution of 5144  ×  3599 pixels and 300 dpi. For each species, specimens were selected to cover a range of typical herbarium material, including broken over folded leaves, leaves damaged by herbivores, and overlapping leaves. The most common German trees include several species that have similar leaves, for example, *Acer plantanoides*, *Acer pseudoplantanus*, *Populus tremula, Populus nigra*, *Quercus cerris*, *Quercus robus*, *Quercus petraea*, *Ulmus glabra*, and *Ulmus minor*. A selection of our 260+ images is available as online supporting material. We initially tried using images from JSTOR but finding 10 per species proved extremely time-consuming and not possible for all 26 species.

### Preprocessing

To extract leaf characteristics from herbarium specimens, the first step is to locate and segment single leafs. The automatic segmentation routines had to distinguish leaves, fruits, flowers and stems, and to cope with overlapping and damaged leaves. We achieved this using the lazy snapping routine [10], which requires that the user exemplarily mark a few points on the leaf and on the background. Lazy Snapping is based on graph cuts and provides visual feedback to the user so that segmentation results can be corrected if necessary. Although fully automatic segmentation routines have been proposed [11], the semi-automatic approach allowed for efficient and flexible processing and was able to deal with overlapping and damaged leaves.

### Normalization

**Fig. 1:**
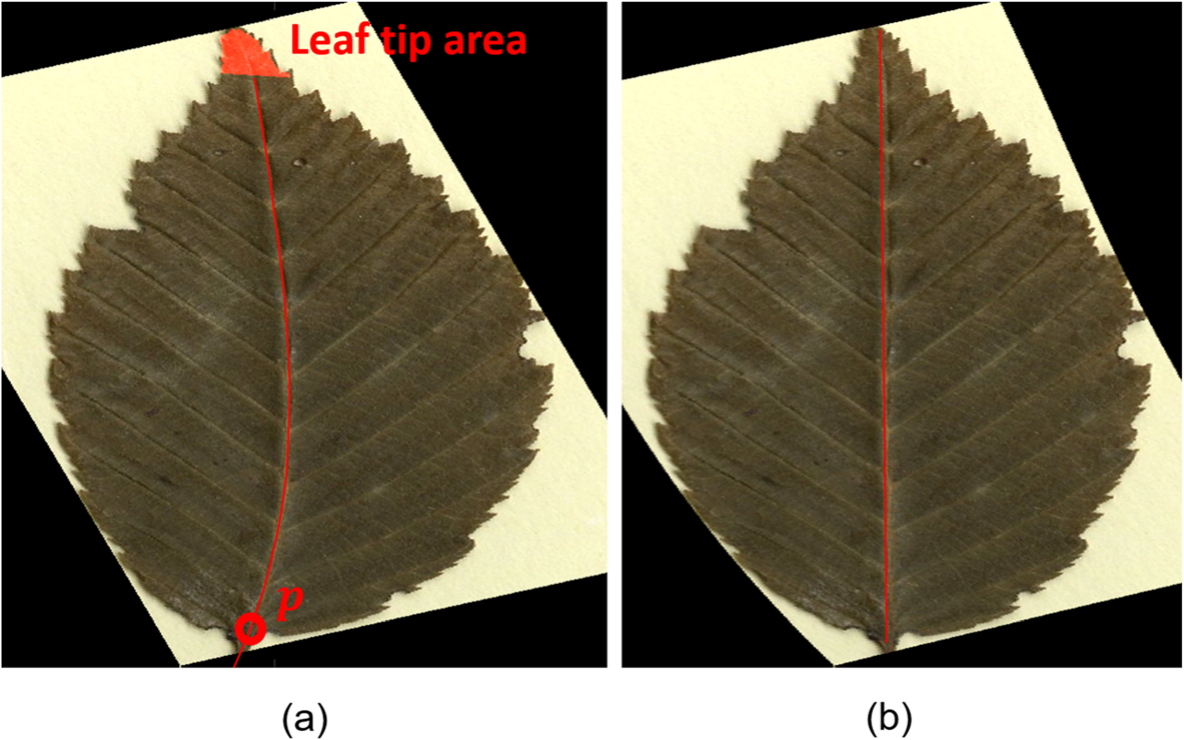
Normalization of leaf distortions: The main vein is detected (**a**) and mapped to a straight line (**b**) providing a consistent data set for classification purposes

**Fig. 2:**
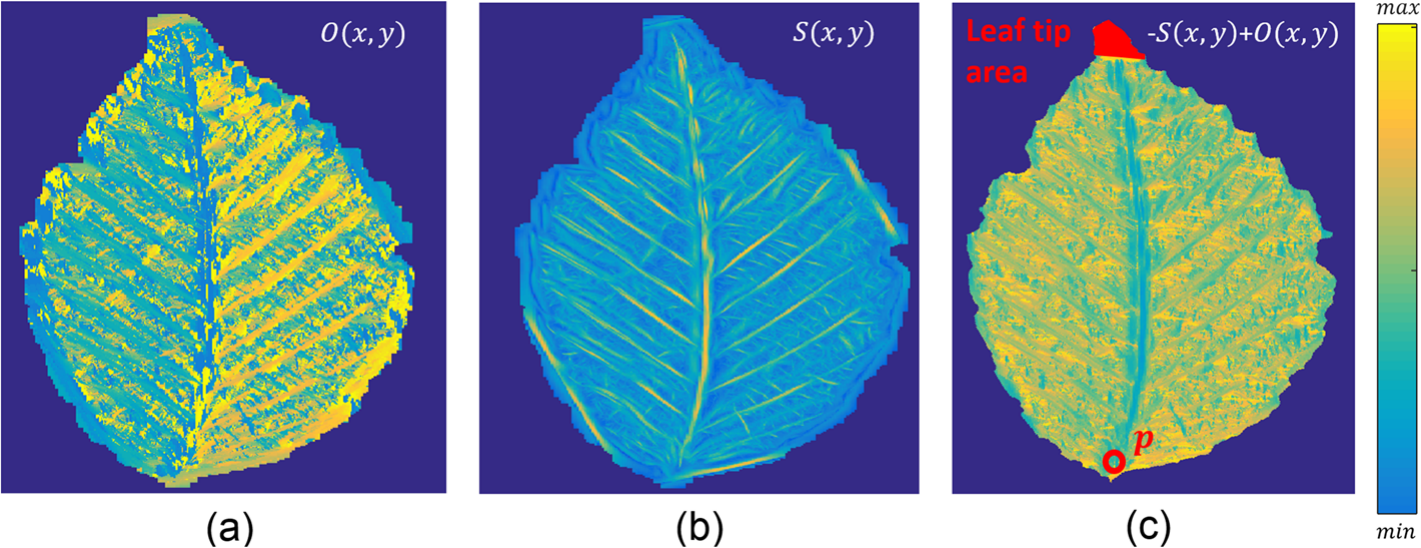
The orientation (**a**) and strength (**b**) of the vein network given by the line operator. The path minimizing ε_*AB*_ from the petiole (marked by a *red p* in **c**) to the leaf tip finally corresponds to the main vein

To counteract shape distortions, the main vein connecting a leaf’s base and apex was aligned to a straight line as illustrated in Fig. 1. To enhance line structures, we applied the line operator described by Zwiggelaar et al. [12], providing line orientation *O*(*x, y*) (Fig. 2a) and strength *S*(*x, y*) (Fig. 2b) measures for each position (*x, y*). Basically, the method matches a line template where the line is passing through the center pixel. Lines of arbitrary orientation are detected by rotating the pattern. The best match determines the strength and orientation, using Gaussian smoothing and subsampling. In our setup, we employed 24 orientations and three subsampling steps using line templates with a length of 15. The main vein was considered as a path from the leaf base to the leaf tip that maximized the line strength and minimized the angle ∆*α*(*x, y*) between the orientation angle and the straight line connecting petiole and leaf tip. A geodesic time algorithm finds a path connecting the leaf base and tip and minimizing ε_*AB*_ = −*S*(*x, y*) + ∆*α*(*x, y*), (1) where the leaf tip is given by the outermost 2% of the segmented area when following the straight line from the petiole passing the centroid of the leaf segmentation (Fig. 2c). This minimization takes into account that the main vein is only slightly curved and points towards the tip of the leaf. The resulting path can be approximated by a third order polynomial (Fig. 1). For a consistent alignment, the leaves were rotated so that *g* was a vertical axis and the leaf tip pointed upwards. Finally, for each row of the image, a horizontal alignment was made so that the identified main vein formed a straight vertical line.

### Feature extraction

Three sets of descriptive features (FS1, FS2, FS3) were defined and served as input to a Support Vector Machine characterizing the leaf shape and leaf veins. Fourier descriptors can easily be modified to be invariant under translation, rotation, and scaling. The first feature set, FS1, consisted of Fourier descriptors characterizing the outline of the binary leaf segmentation. The second set, FS2, consisted of the descriptive leaf shape parameters compactness, convexity, solidity, rectangularity, circularity, perimeter-area ratio, slimness, position of maximum thickness, and dispersion [13, 14]. The third set, FS3, quantified the structure of the vein network. Because a pixel-wise identification of the vein network could not be performed in a robust manner, we focused on features representing the orientation of the vein structure by using weighted orientation histograms.

**Fig. 3:**
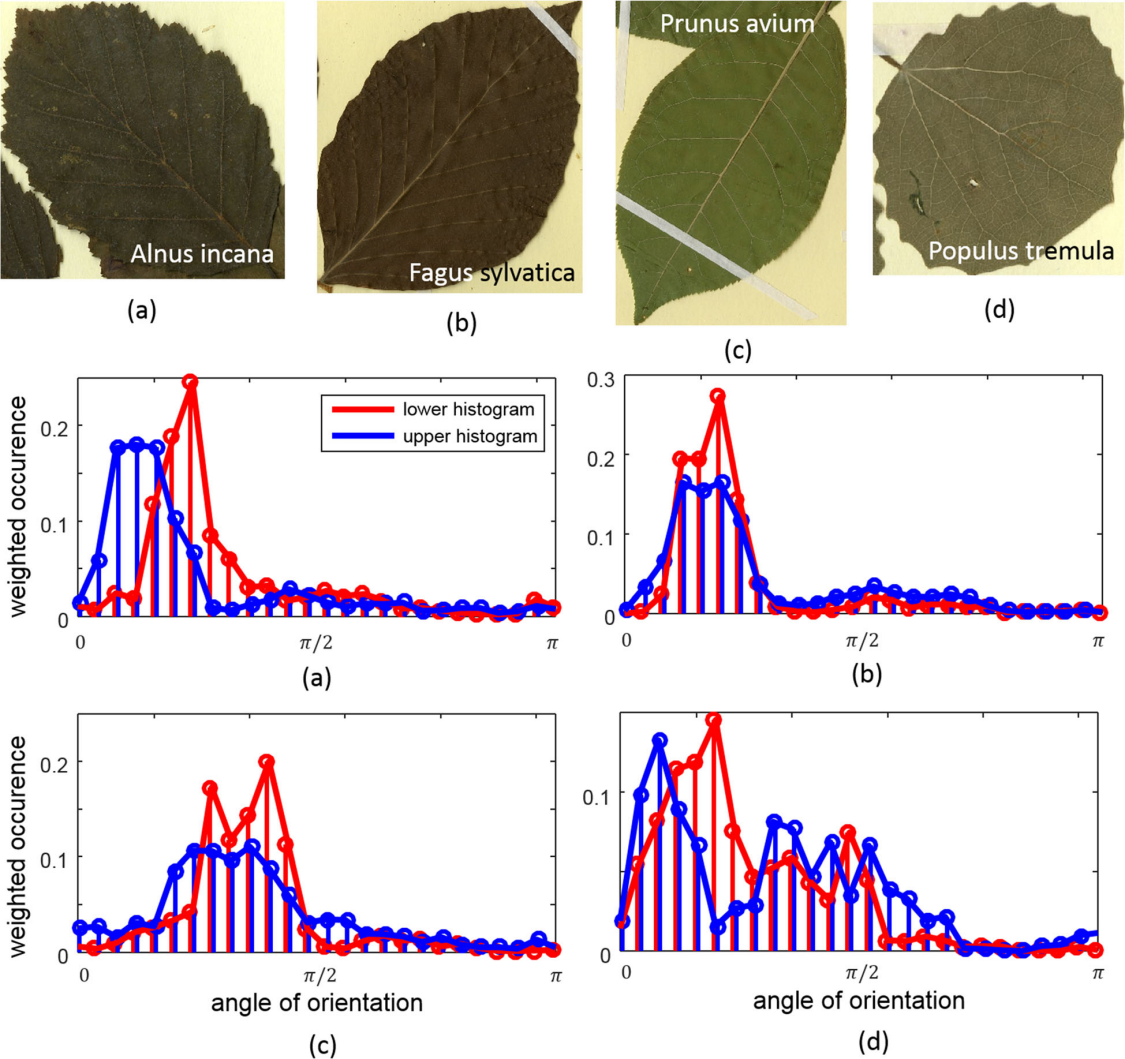
Examples for leaf species (*upper row*) and their corresponding weighted histograms (*lower rows*) characterizing their leaf vein network

Figure 3 illustrates examples for leaf species (upper row) and their corresponding weighted histograms (lower rows) characterizing their leaf vein network. In weighted histograms, each pixel is weighted by its line strength, and the cumulative weight of each of the 24 orientation bins is presented. The histograms were evaluated for the upper and lower half of each leaf and averaged for the left and the right side. Different vein networks are indicated by the peak locations in the histograms as well as the spread and shifts between upper and lower peaks. For example, although the vein networks of *Alnus incana* and *Fagus sylvatica* are optically similar, their histograms emphasize different vein angle signatures. *Alnus incana* (Fig. 3a) shows a shift of the orientation angle between lower and upper parts of the leaf, whereas *Fagus sylvatica* (Fig. 3b) has parallel venation. The histograms therefore can serve as fingerprints of the venation networks and constitute the vein network-related feature set.

### Classifier setup

A Support Vector Machine (SVM) was trained in order to assign unknown feature vectors to one of the species classes. The SVM was configured with a linear kernel providing the best performances. Due to the low number of mages per species, a leave-one-out validation strategy was pursued. Multiclass classification was realized through the one-against-one strategy [15].

### Validation

Two test sets were used for validation. Test set I consisted of all 26 species (Additional file 1: Table S1), a few of them in the same genus. In contrast, test set II included only one species per genus, leaving 17 species.

## Results

**Fig. 4:**
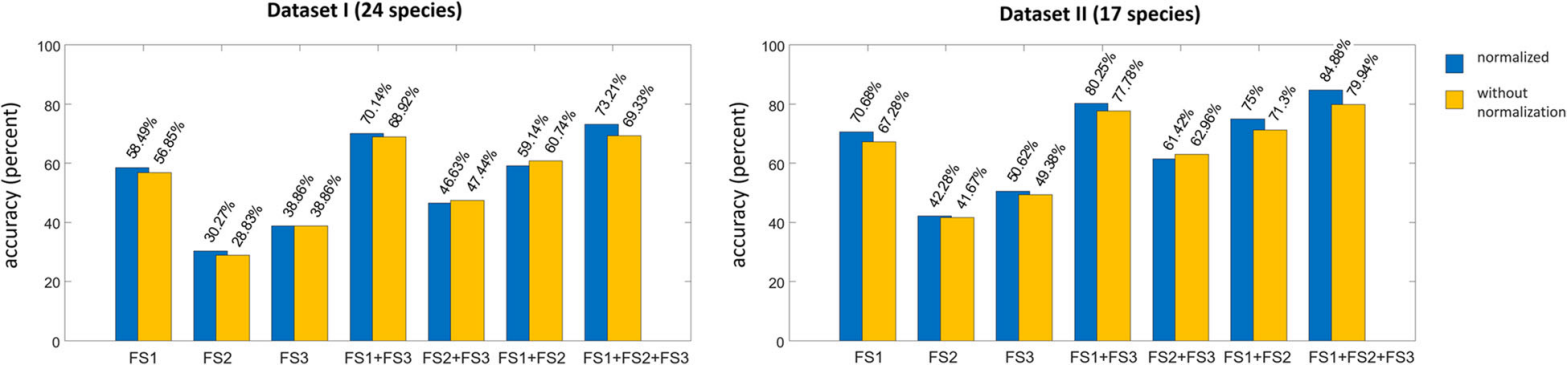
Classification accuracy obtained with data sets I and II and the feature sets FS1, FS2, FS3 and combinations thereof. The best classification results are observed for a combination of all three feature sets combined with the proposed normalization step

**Fig. 5:**
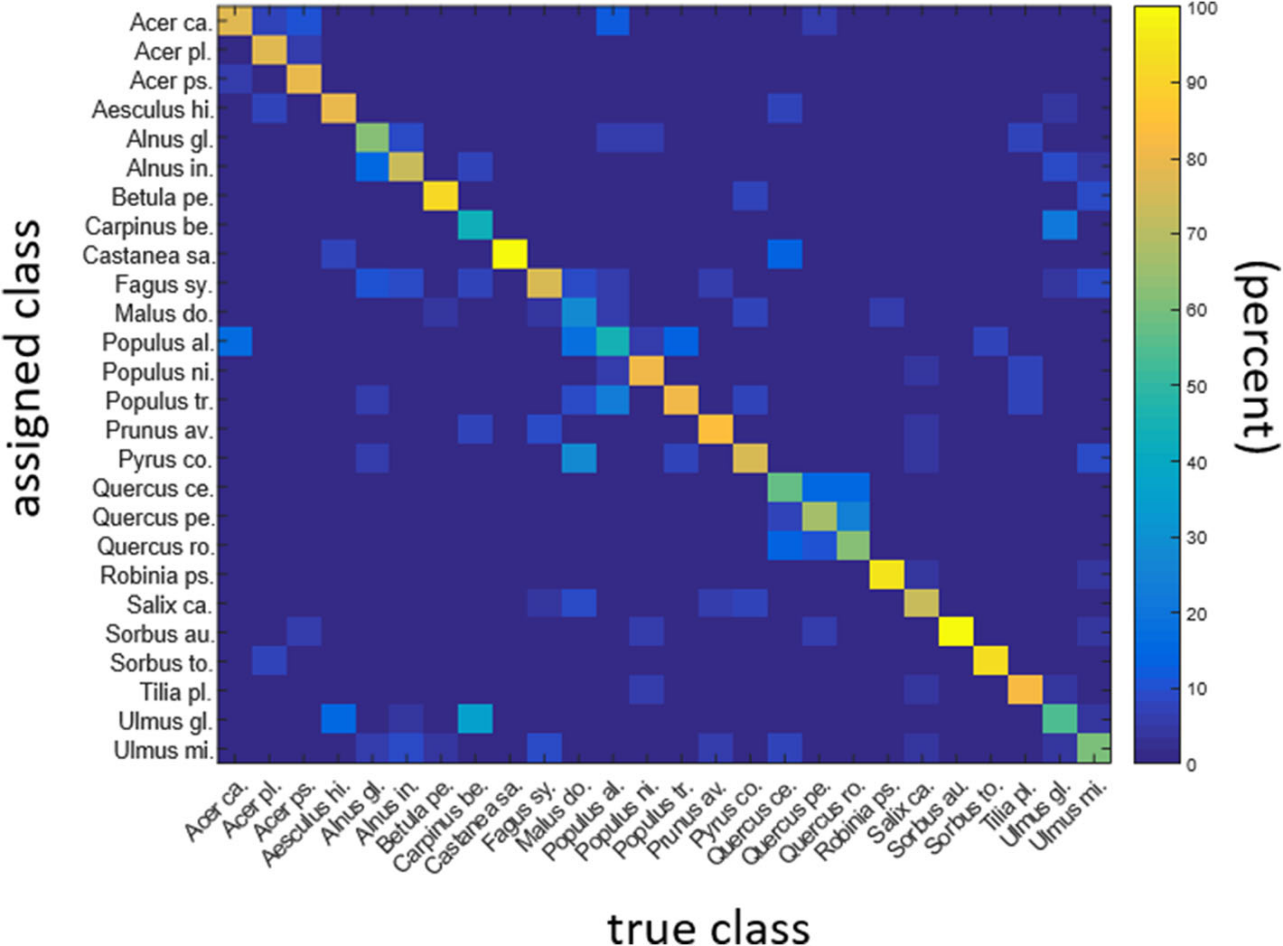
Confusion matrix showing the true class (*x axis*) and the class assigned by the system (*y axis*), at the 10-image minimum per category. The color-coding is explained to the right and refers to the percentage correctly identified

**Fig. 6:**
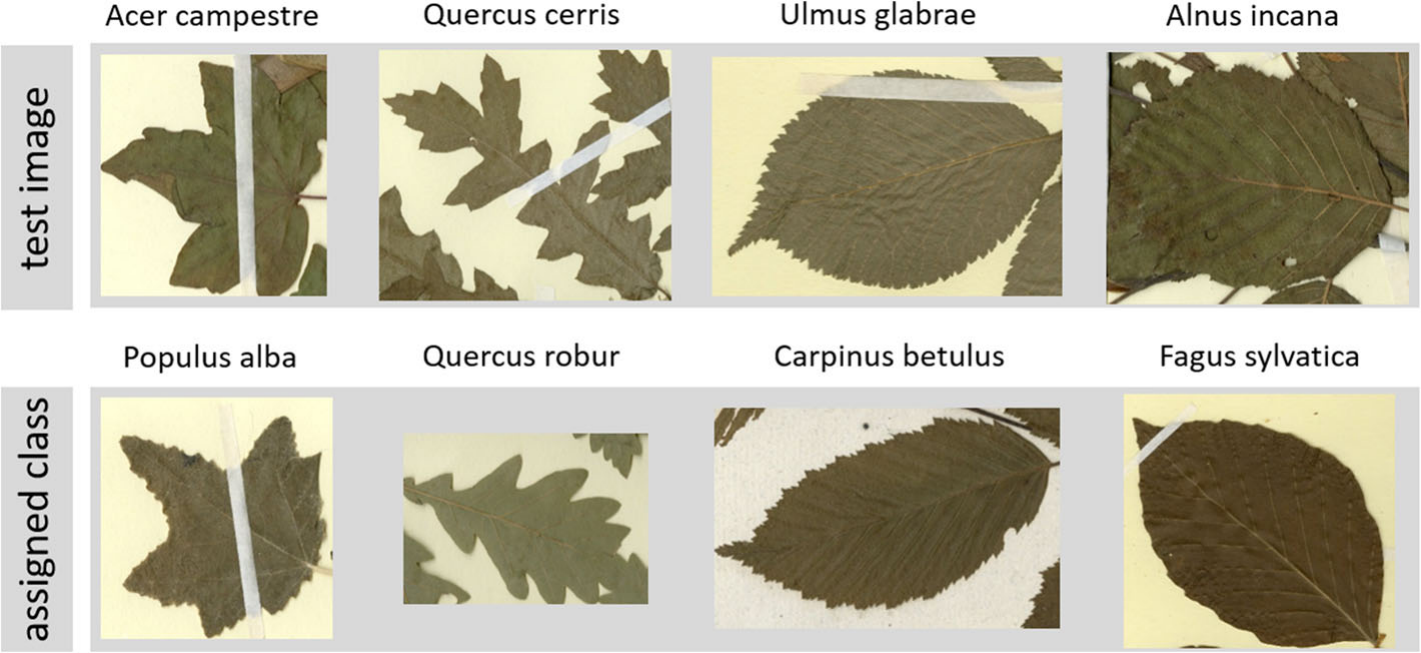
True species assignments in the first row; assignment achieved by the system in the second. Morphologic similarities (especially between *Acer plantanoides*, *Acer pseudoplantanus*, *Populus tremula, Populus nigra*, *Quercus cerris*, *Quercus robus*, *Quercus petraea*, *Ulmus glabra*, and *Ulmus minor*) and damaged leaves caused misclassifications

The number of Fourier descriptors was set to 20, which was found to perform best in both test sets. With regard to the individual feature sets (FS1, FS2, FS3), the best overall performance was otained when all three were combined. With this approach, we achieved 73.21% of accuracy in test set I, and 84.88% in test set II (Fig. 4). The normalization step, which straightened the midvein, considerably increased the classification accuracy as seen in in Fig. 4, where the yellow bar shows the results without the normalization step and the blue bar with this step. This was particularly true for the Fourier descriptors and the combination of the three feature sets. Figure 5 shows the confusion matrix of test set I (26 species of common German trees, each represented by 10 images of herbarium specimens) when the three feature sets were combined. Figure 6 shows examples of frequent misclassifications.

## Discussion

Taxonomists are increasingly relying on digitized images, either to achieve specimen identification via visual matching of features or to extract morphological features that can be coded and used for phylogenetic, morphometric, or other purposes [9, 11, 16, 17]. Because of the great number of databased images now available and the comparatively few taxonomic experts, there is a great need for computer vision to be applied to specimen images of which millions are being made available online at substantial costs ([16]; cf. our Introduction with data on JSTOR images and usage numbers). Deep learning approaches [18] for computer vision in principle could allow automated plant specimen identification --meaning the suggestion of a Latin name for the respective image-- as long as the software could be trained on suitable subsets of the millions of Latin-named plant images already available online. Extracting such subset is not an easy task, however, and the first insight from this project was that we had to make new images (using the HerbScan setup applied in many herbaria for specimen digitization) to obtain 10 images for each of the tree species. It is an empirical fact that most of the estimated 340,000 species of higher plants are known from few collections and are so far represented by few images in public databases. Therefore the use of 10 images for training purposed sets a realistic bar.

Our success rate of 73 to 85% with the two test sets is comparable to that in the few other projects that have applied computer vision to name plants by clustering of similar leaf types, although not necessarily on finding *formal [Latin] species names*. For example, the Leafsnap application developed by Kumar et al. [19], which identifies common North American tree species in the Washington (District of Colombia) area, had success rates of 96.8%, but requires snapshots of fresh, non-overlapping leaves. A classification of different grapevine varieties (from photos of perfectly spread out fresh leaves) had success rates of up to 80% [13]. Scans of herbarium specimens of four fern species, using 18 scans per species, gave classification accuracies of at least 96% [20]. We found not data on identification success rates for image sharing and retrieval applications, such as Pl@ntNet, where users can upload photos of plants and identify them for others ([21] for a critique). For comparison to all these photo-based tools, a study in which 67 common British trees (which includes the common ‘German’ species) were identified by barcoding had species discrimination success rates of 65 to 86% [22].

## Conclusions

This study represents the first application of computer vision to images of old herbarium specimens, similar to the 2.48 million specimen images in JSTOR. There is a need to efficiently use images of herbarium material (all with scientific [Latin] species names) that are stored in public databases by making them more useful for non-specialists who need names for their plants. The results demonstrate that computer vision can be used to classify specimens even when they have many overlapping leaves and even when few training images are available. Rapid identification even just to the genus level could help non-botanists who need a quick list of the tree species in a particular street or park.

## Additional file

Additional file 1: Table S1. The 26 tree species most common in Germany used on this study. (DOC 58 kb)
