# Supplementary material for: Computer vision applied to herbarium specimens of German trees: testing the future utility of the millions of herbarium specimen images for automated identification
Source: BMC Evol Biol. 2016 Nov 16;16:248. doi: 10.1186/s12862-016-0827-5 (PMC5112707; doi:10.1186/s12862-016-0827-5)
Supplement: Additional file 1: Table S1. — The 26 tree species most common in Germany used on this study. (DOC 58 kb) [file 12862_2016_827_MOESM1_ESM.doc]

**Table S1** The 26 tree species most common in Germany used on this study.

Data set I

*Acer campestre* (Feldahorn)

*Acer platanoides* (Spitzahorn)

*Acer pseudoplatanus* (Bergahorn)

*Aesculus hippocastanum* (Rosskastanie)

*Alnus glutinosa* (Schwarzerle)

*Alnus incana* (Weißerle)

*Betula pendula* (Birke)

*Carpinus betulus* (Hainbuche)

*Castanea sativa* (Edelkastanie)

*Fagus sylvatica* (Buche)

*Malus domesticus* (Apfel)

*Populus alba* (Silberpappel)

*Populus nigra* (Schwarzpappel)

*Populus tremula* (Zitterpappel)

*Prunus avium* (Vogelkirsche)

*Pyrus communis* (Birne)

*Quercus cerris* (Erreiche)

*Quercus petraea* (Traubeneiche)

*Quercus robur* (Stieleiche)

*Robinia pseudoacacia* (Robinie)

*Salix caprea* (Salweide)

*Sorbus aucuparia* (Eberesche)

*Sorbus torminalis* (Elsbeere)

*Tilia platyphyllos* (Sommerlinde)

*Ulmus glabra* (Bergulme)

*Ulmus minor* (Feldulme)

Data set II

*Acer pseudoplatanus* (Bergahorn)

*Aesculus hippocastanum* (Rosskastanie)

*Alnus glutinosa* (Schwarzerle)

*Betula pendula* (Birke)

*Carpinus betulus* (Hainbuche)

*Castanea sativa* (Edelkastanie)

*Fagus sylvatica* (Buche)

*Malus domesticus* (Apfel)

*Populus nigra* (Schwarzpappel)

*Prunus avium* (Vogelkirsche)

*Pyrus communis* (Birne)

*Quercus petraea* (Traubeneiche)

*Robinia pseudoacacia* (Robinie)

*Salix caprea* (Salweide)

*Sorbus aucuparia* (Eberesche)

*Tilia platyphyllos* (Sommerlinde)

*Ulmus minor* (Feldulme)
